# Regional changes in cerebral blood flow between the upright and supine posture and over 3 days of bed rest

**DOI:** 10.1113/EP091820

**Published:** 2025-01-22

**Authors:** Carmen Possnig, Kyohei Marume, Gautam Babu, Sylvan L. J. E. Janssen, Christopher M. Hearon, Katrin A. Dias, Satyam Sarma, Justin S. Lawley, Benjamin D. Levine

**Affiliations:** ^1^ Department of Sports Science, Performance Physiology & Prevention University of Innsbruck Innsbruck Austria; ^2^ Institute for Exercise and Environmental Medicine Texas Health Presbyterian Dallas Dallas Texas USA; ^3^ University of Texas Southwestern Medical Center Dallas Texas USA; ^4^ University Medical Center Radboud University Nijmegen The Netherlands; ^5^ Institute of Mountain Emergency Medicine Eurac Research Bolzano Italy

**Keywords:** bed rest, cerebral blood flow, cerebral hypoperfusion, microgravity, posture changes, regional heterogeneity

## Abstract

A reduction in cerebral blood flow (CBF) has been observed during spaceflight and bed rest. We aimed to examine the magnitude and regional heterogeneity of the decrease in CBF during bed rest compared to posture changes on Earth. Seventeen participants (age, 29 ± 9 years, 7 females) were studied in the upright and supine posture and over 3 days of bed rest. We assessed blood flow via duplex ultrasonography in the internal carotid (ICA) and vertebral arteries (VA), and via transcranial Doppler of the middle cerebral artery (MCAv). Mean arterial pressure (MAP) and end‐tidal CO_2_ ($ET_{CO_{2}}$) were assessed at all time points. By day 3, total CBF (1078 ± 302 to 853 ± 245 mL min^−1^ *, P <* 0.0001) and MCAv (61 ± 15 to 49 ± 12 mL min^−1^, *P <* 0.0001) were decreased compared to the supine posture. CBF values did not fall below the upright posture (all *P* > 0.05) but were lower than a calculated 24‐h mean baseline (*P* = 0.0132). MAP remained stable (*P =* 0.971), as did $ET_{CO_{2}}$ (*P* = 0.0803), while VA blood flow decreased after 24 h and again after 72 h (*P* = 0.0024). These findings indicate that CBF decreases during short‐term bed rest, but not below values observed in the upright posture.

## Abstract (highlights)

- **What is the central question of this study?**
  Does cerebral blood flow change with bed rest and if yes, are there any regional heterogeneities and what are the underlying mechanisms?
- **What is the main finding and its importance?**
  Cerebral blood flow decreases over bed rest involving both the internal carotid and vertebral artery blood flow. While it does not fall below values in the seated position, it is lower than a 24‐h mean calculated from both seated and supine baseline values. End‐tidal CO_2_ and mean arterial pressure are not likely to be mechanisms for the decrease, as they did not change accordingly.

## 1 INTRODUCTION

As humanity prepares to return to the Moon and long‐duration spaceflights to Mars are envisioned, studying the effects of microgravity on the human body is evermore in focus. Opportunities to perform research directly on astronauts in space, however, are rare. One model to simulate microgravity is to perform experiments with participants resting in a horizontal or −6° head‐down tilt (HDT) position. These Earth‐bound bed rest studies can reproduce some of the effects of microgravity. As the *G_z_* gravitational vector is eliminated during bed rest, fluid shifts similar to those in microgravity are observed. This experimental set‐up adequately represents spaceflight‐induced changes in the cardiovascular system. When studying the cerebral circulation during bed rest, a decrease in cerebral blood flow is a common finding. For instance, a study using phase‐contrast magnetic resonance imaging revealed that, relative to the supine position, cerebral blood flow is reduced after only 4.5 h of −6° HDT bed rest (Marshall‐Goebel et al., 2016). Similar results can be found over at least 30 days of bed rest in studies using ultrasonography and magnetic resonance imaging to investigate cerebral blood flow (Kramer et al., 2017; Ogoh et al., 2020; Roberts et al., 2021). In contrast, another study found no differences in blood flow to the brain after 21 days of bed rest, albeit assessing cerebral blood flow indirectly via middle cerebral artery velocity (MCAv) (Jeong et al., 2014). However, a noteworthy limitation of these previous studies is a lack of context relative to normal daily fluctuations in cerebral blood flow on Earth. In the course of daily life on Earth, cerebral blood flow changes according to posture, whereby cerebral perfusion is lower in the upright versus supine position (Alperin et al., 2005; Sato, Fisher, et al., 2012). As humans spend most of their awake time upright, the question arises whether the decrease in cerebral blood flow with bed rest falls below that typically observed in the upright posture.

The brain, with its extraordinarily high metabolic demand, has very limited energy storage and does not tolerate reductions in oxygen delivery well (Bangen et al., 2014; Benedictus et al., 2017; Lassen, 1974; Leeuwis et al., 2017). Indeed, reduced cerebral blood flow has been linked to cognitive impairments (Ruitenberg et al., 2005; Wolters et al., 2017). Evidence for cognitive impairment has also been seen during experimental bed rest (Lipnicki et al., 2009; Liu et al., 2012) and during hospitalization, which may be seen as a real‐world application of bed rest (Calero‐García et al., 2017). In order to rule out a risk for cognitive impairment in astronauts on long‐duration flights, determining the magnitude of the observed reduction in cerebral blood flow during bed rest and comparing it to both supine and seated values is an important step toward safety in space exploration.

An additional limitation to previous studies noting a fall in cerebral blood flow during bed rest is the lack of concurrent physiological variables that regulate cerebral blood flow, such as cerebral perfusion pressure (CPP), cerebral metabolism (the rate of cerebral oxygen uptake), neural sympathetic control, arterial concentration of CO_2_ and O_2_, and cerebral autoregulation (Willie et al., 2014). In the context of changes in posture, bed rest and/or normoxic spaceflight, the primary factors regulating cerebral blood flow are likely CPP and the arterial concentration of CO_2_. The steady‐state nature of bed rest likely limits the role of CPP because (1) static autoregulation is likely intact in healthy individuals and (2) CPP at brain level is similar despite changes in posture due to concurrent changes in both intracranial pressure and blood pressure at brain level (Petersen, Petersen, et al., 2016). Changing posture from the seated to the supine position is accompanied by hypocapnia, alongside a decrease in CBF (Serrador et al., 2006). Thus, it seems the arterial concentration of carbon dioxide is a primary candidate for decreases in cerebral blood flow with changes in posture, and should be investigated during bed rest.

Therefore, the overall aim of this study was to document the time course of changes in cerebral blood flow over 3 days of bed rest and contrast these to both the upright and supine posture. Our first hypothesis was that the decrease in cerebral blood flow observed by previous studies would not fall below the seated baseline values.

Moreover, to determine concurrent changes in two mechanistic candidates, the end‐tidal concentration of CO_2_ and mean arterial blood pressure were measured concurrently. Previously it has been shown that the posterior cerebral circulation has a blunted reactivity to carbon dioxide (Sato, Sadamoto, et al., 2012). Moreover, during changes in posture, blood flow in the vertebral artery remains unchanged despite a decrease in blood flow through the internal carotid artery (ICA) (Ogoh et al., 2016). Therefore, a secondary aim of the study was to examine potential regional differences in cerebral blood flow during bed rest. We hypothesized that blood flow through the ICA would decrease whereas blood flow through the vertebral artery would remain stable during bed rest.

## 2 METHODS

### 2.1 Ethical approval

All participants were informed of the purpose and risks of each procedure and signed an informed consent form, which was approved by the Institutional Review Board at the University of Texas Southwestern Medical Center and followed guidelines set forth in the *Declaration of Helsinki* (STU102015‐057).

### 2.2 Participants

Ten men (age, 31 ± 10 years; height, 177 ± 10 cm; weight, 75 ± 20 kg) and seven women (age, 26 ± 5 years; height, 164 ± 7 cm; weight, 71 ± 19 kg) took part in this study. All participants were healthy, non‐smokers free from cardiovascular and neuromuscular diseases and not taking any medication (including contraceptives). Females were studied in the self‐reported early follicular phase of their menstrual cycle (days 1–7) (Sims & Heather, 2018). Participants refrained from doing strenuous exercise or drinking beverages containing alcohol or caffeine 24 h before the study. Food and caffeine intake were not controlled during the period of bed rest but were not consumed for ∼12 h (i.e., an overnight fast) before measurement time points.

### 2.3 Experimental procedures

#### 2.3.1 Protocol

**FIGURE 1:**
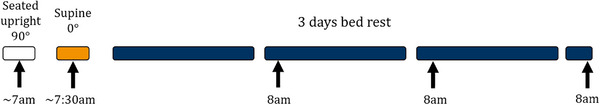
Systematic overview of study protocol from the arrival of the participant (07.00 h on day 1) to end of bed rest 72 h later (08.00 h on day 4) Measurements taken (arrows): sonography (middle cerebral artery, vertebral artery and internal carotid artery), end‐tidal CO_2_ and cardiovascular parameters.

All trials were completed in a quiet, climate‐controlled environment in the Clinical Translational Science Center at The University of Texas Southwestern Medical Center. Participants were instrumented in a seated posture. After 20 min of sitting in the 90° upright posture, 5 min of steady‐state haemodynamics were obtained. Thereafter, the subject was transferred to a supine position and after 20 min, the same measurements were repeated. This baseline testing was followed by 3 days of strict bed rest. This paper combines participants from two slightly different protocols, whereby five men and two women underwent bed rest in a −6° HDT position and five men and five women underwent bed rest in a 0° supine position, which was defined by the National Aeronautics and Space Administration (NASA) standard at the time. The two studies were separated by a 1‐year interval. In both set‐ups, cardiorespiratory parameters and ultrasonography measurements were obtained at 08.00 h each morning (Figure 1). Throughout the entire bed rest protocol, participants were monitored to assure strict adherence to the −6° or supine position without any head elevation (e.g., pillow use).

#### 2.3.2 Cardiorespiratory parameters

##### Heart rate

Continuous recordings of heart rate were determined from a three‐lead electrocardiogram (Tram‐rac, Solar 8000M, GE Healthcare, Marquette, MI, USA).

##### End‐tidal carbon dioxide

Exhaled carbon dioxide was sampled continuously by capnography (Novametrix, Criticare Systems, Milwaukee, WI, USA) from a nasal cannula for determination of end‐tidal CO_2_ ($ET_{CO_{2}}$).

##### Arterial blood pressure

Arterial blood pressure was measured in duplicate (and averaged) by electrosphygmomanometry (SunTechMedical Instruments Inc., Durham, NC, USA) with a microphone placed over the brachial artery to detect Korotkoff sounds.

#### 2.3.3 Total cerebral arterial blood flow

##### Ultrasonography

The right internal carotid and VA were imaged using a 12‐MHz linear‐array Doppler probe (model M12L; Vivid 7, GE Healthcare, Milwaukee, WI, USA). The duplex mode was used to measure the time‐averaged mean blood flow velocity via a Doppler audio transformer (pulse‐wave mode with 60° insonation angle) and, at the same time, artery diameter (B‐mode) over 30 s (Herr et al., 2010; Thomas et al., 2015). Continuous screen capture (Epiphan Systems Inc., California and Camtasia Studio, TechSmith, Okemos, MI, USA) and offline analysis of the artery diameter was carried out using automated wall tracking software (Brachial Analyzer, Medical Imaging Applications LLC, Coralville, IA, USA). Assuming bilateral symmetry of the internal carotid and vertebral arteries, total cerebral blood flow was calculated as the product of the right ICA and VA mean blood velocity (cm s^−1^) and artery cross‐sectional area (cm^2^) multiplied by 2 and expressed as millilitres per minute.

MCAv was continuously recorded during testing time points using transcranial Doppler ultrasound (Multigon Industries, Yonkers, NY, USA). The right middle cerebral artery was located as follows. Initially, the sample volume was placed at a depth of 55 mm while performing a transcranial searching pattern. Once the middle cerebral artery was identified, simultaneous multi‐depth Doppler and M‐modes were used to scan its length and identify the optimal depth based on signal morphology and blood velocity. At this location, the probe was securely attached to the cranium by a mould that was cast individually to fit the ear structure (Giller & Giller, 1997), to maintain both probe position and angle during repeated testing. The cerebral vascular conductance of each artery was calculated as the ratio of the mean blood flow or velocity (expressed as index) and mean arterial pressure (MAP).

All cardiovascular variables were recorded as analog data at 250 Hz and transferred to a laptop using a standard AD converter (MP150, Biopac, Goleta, CA, USA).

#### 2.3.4 Statistical analysis

To examine the effect of bed rest, data were analysed using a single factor (time: upright, supine, and bed rest day 1, day 2 and day 3) repeated measures analysis of variance with the following questions formulated a priori and including *post hoc* follow up comparisons (Holm–Šídák): (1) normal gravitation gradients on Earth: upright versus supine; (2) the time course of changes in cerebral blood flow over 3 days of bed rest: supine posture versus bed rest day 1, day 2 and day 3; (3) do 3 days of bed rest cause a reduction in cerebral blood flow below the normal upright posture on Earth? Upright versus bed rest day 3. All values are expressed as means ± standard deviation. All statistical procedures were completed on Prism version 6 (GraphPad Software, Boston, MA, USA) where statistical significance was set to *P* ≤ 0.05.

#### 2.3.5 Power calculation

A previous study by Marshall‐Goebel et al. (2016) noted a statistically significant decrease in total brain blood flow (∼15%) over 4.5 h of HDT. Based on these data and the measurement of the internal carotid and vertebral blood flow in our hands, we modelled that between 6 and 20 participants would be needed to observe a 15–20% decrease in internal carotid ((effect size) ES = 1.0–1.42) and vertebral (ES = 0.45–0.67) artery blood flow with a power of 80% at an α level of 0.05.

## 3 RESULTS

### 3.1 Changes in cerebral blood flow after the transition from an upright to a supine posture on Earth

**FIGURE 2:**
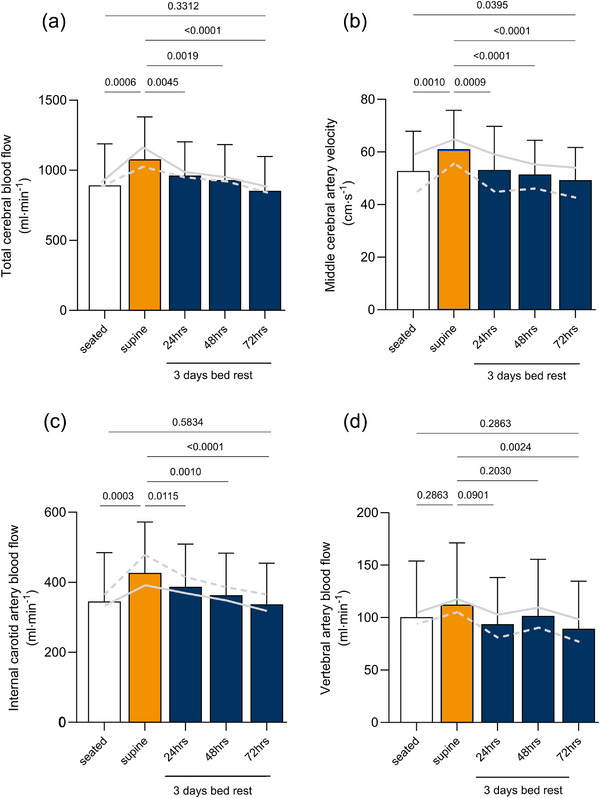
Results of baseline measurements and 3 days of bed rest. *P*‐values during bed rest are all compared to supine posture, as no differences were observed between 72 h of bed rest and the upright posture. (a) Total cerebral blood flow significantly increased from seated to supine position, then consequently decreased over the bed rest phase (*P *< 0.001). (b–d) MCAv (b) and ICA blood flow (c) decreased in a similar fashion after 72 h (*P* < 0.001) while vertebral artery blood flow did not change significantly (d) from seated to supine position, and showed a significant decrease during bed rest only after 72 h (*P* = 0.0024). Bars: all participants (*n* = 17) included. Dashed grey line: −6° head down tilt protocol (*n* = 7); continuous grey line: supine protocol (*n* = 10). ICA, internal carotid artery; MCAv, middle cerebral artery velocity.

**FIGURE 3:**
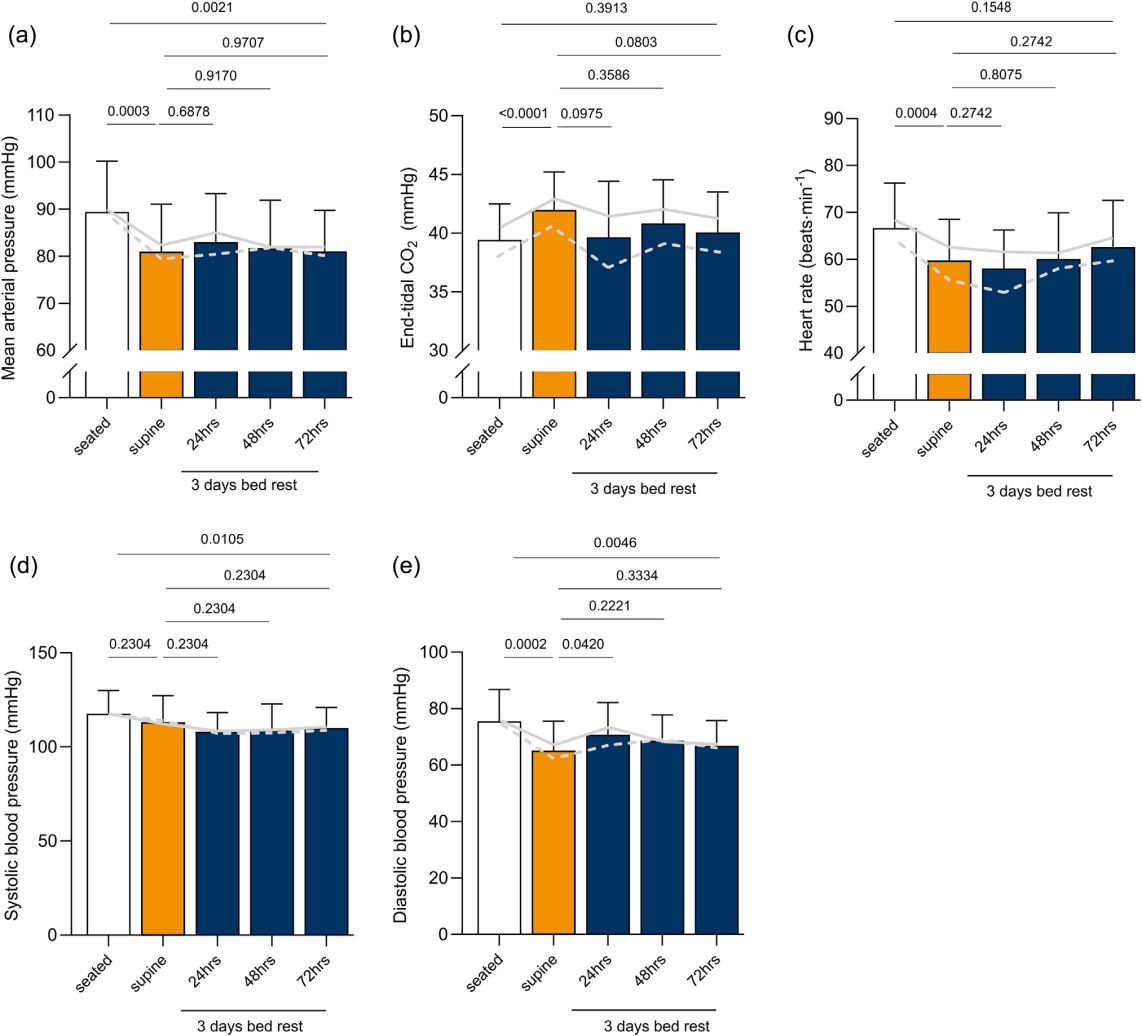
Results of haemodynamics and $ET_{CO_{2}}$ measurements. *P*‐values during bed rest are all compared to supine posture. (a) MAP decreased significantly from the seated to supine position (*P* < 0.001), then remained relatively constant during bed rest. (b) $ET_{CO_{2}}$ increased from seated to supine (*P* < 0.001), then did not decrease significantly during bed rest. (c–e) Heart rate decreased with movement from seated to supine (c), while systolic blood pressure showed no significant changes in any posture (d), and diastolic blood pressure decreased from seated to supine posture and increased slightly (e) during the first 24 h of bed rest. Bars: all participants (*n* = 17) included. Dashed grey line: −6° head down tilt protocol (*n* = 7); continuous grey line: supine protocol (*n *= 10). $ET_{CO_{2}}$, end‐tidal CO_2_; MAP, mean arterial pressure.

Total cerebral blood flow was higher in the supine compared to the upright posture by 17.3% (seated vs. supine, 891 ± 296 vs. 1078 ± 302 mL min^−1^; *P* = 0.0006, Figure 2). The increase in total cerebral blood flow was mostly due to changes in blood flow to the anterior cerebral circulation, as ICA blood flow (345 ± 140 vs. 427 ± 146 mL min^−1^; *P* = 0.0003, Figure 2) as well as MCAv (53 ± 15 vs. 61 ± 15 cm s^−1^; *P* = 0.001, Figure 2) increased. In contrast, blood flow in the vertebral artery remained unchanged (100 ± 54 vs. 112 ± 59 mL min^−1^; *P* = 0.2863, Figure 2). $ET_{CO_{2}}$ was higher in the supine compared to the seated position (seated vs. supine, 39 ± 3 vs. 42 ± 3 mmHg; *P* < 0.0001, Figure 3), and mean arterial blood pressure, measured at the brachial artery, increased significantly with the change in posture to the upright position (89 ± 11 vs. 81 ± 10 mmHg; *P* = 0.0004, Figure 3). An overview of haemodynamic parameters in the upright seated and supine position is presented in Table 1.

**TABLE 1:** Systemic and cerebral haemodynamic parameters before (baseline), during (24 and 48 h) and after (72 h) bed rest.

Parameter	Baseline		3 days’ bed rest		
	Seated 90°	Supine	24 h	48 h	72 h
Total cerebral blood flow (ml min^−1^)	891 ± 296^**^	1078 ± 302	962 ± 241^*^	930 ± 253^*^	853 ± 245^**^
Cerebrovascular conductance (mL min^−1^ mmHg^−1^)		13.55 ± 4.37	11.66 ± 2.8^*^	11.61 ± 3.88^*^	10.71 ± 3.6^**^
MCA velocity (cm s^−1^)	53 ± 15*	61 ± 15	53 ± 16^**^	52 ± 13^**^	49 ± 12^**^
MCA conductance index (cm s^−1^ mmHg^−1^)		0.77 ± 0.23	0.65 ± 0.22^**^	0.64 ± 0.21^**^	0.62 ± 0.18^**^
$ET_{CO_{2}}$ (mmHg)	39 ± 3^**^	42 ± 3	40 ± 5	41 ± 4	40 ± 3
HR (beats min^−1^)	67 ± 10^**^	60 ± 9	58 ± 8	60 ± 10	63 ± 10
MAP (mmHg)	89 ± 11^**^	81 ± 10	83 ± 10	82 ± 10	81 ± 9
SBP (mmHg)	117 ± 12	113 ± 14	108 ± 10	109 ± 14	110 ± 11
DBP (mmHg)	75 ± 11^**^	65 ± 10	71 ± 11^*^	68 ± 9	67 ± 9

*Note*: Values are means ± SD. *n* = 17. *P*‐values of changes are all compared to supine posture. *P < 0.05, **P < 0.001; for detailed *P*‐values see ‘Results’ section and figures. Abbreviations: DBP, diastolic blood pressure; $ET_{CO_{2}}$, end‐tidal carbon dioxide; HR, heart rate; MAP, mean arterial pressure; MCA, middle cerebral artery; SBP, systolic blood pressure.

### 3.2 Changes in cerebral blood flow after 3 days of bed rest

#### 3.2.1 Bed rest versus supine baseline

After 24 h of bed rest, total cerebral blood flow decreased compared to the initial supine measurements (supine vs. 24 h, 1078 ± 302 vs. 962 ± 241 mL min^−1^; *P* = 0.0045, Figure 2) and continued to do so throughout the 3 days, after 72 h reaching a reduction of ∼21% (853 ± 245 mL min^−1^; *P* < 0.0001, Figure 2). Cerebrovascular conductance decreased significantly compared to the supine posture after 24 h (supine vs. 24 h, 13.55 ± 4.4 vs. 11.66 ± 2.8 mL min^−1^ mmHg^−1^; *P* = 0.0038), 48 h (11.61 ± 3.9 mL min^−1^ mmHg^−1^; *P* = 0.0036), and 72 h (10.71 ± 3.6 mL min^−1^ mmHg^−1^; *P* < 0.0001). Examining the regional heterogeneity of blood flow to the brain, ICA blood flow decreased during the first 24 h (427 ± 146 vs. 387 ± 122 mL min^−1^; *P* = 0.0115) and remained below the supine baseline throughout 72 h (all, *P* < 0.001). Conductance in the ICA decreased in a similar way, reaching the minimum after 72 h (supine vs. 72 h, 5.32 ± 1.8 vs. 4.2 ± 1.5 mL min^−1^ mmHg^−1^; *P* < 0.0001). Confirming the relative anterior cerebral hypoperfusion, MCAv showed a similar trend over the 72 h (supine vs. 72 h, 61 ± 15 vs. 49 ± 12 cm s^−1^; *P* < 0.0001). The conductance index in the MCAv fell accordingly (supine vs. 72 h, 0.77 ± 0.23 vs. 0.62 ± 0.18 cm s^−1^; *P* < 0.0001). Blood flow to the posterior circulation was more variable, falling below the supine posture only after 3 days of bed rest (supine vs. 72 h, 112 ± 59 vs. 90 ± 45 mL min^−1^; *P* = 0.0024). Vertebral artery conductance reached a statistically significant decrease only after 72 h (supine vs. 72 h, 1.46 ± 0.9 vs. 1.16 ± 0.7 mL min^−1^ mmHg^−1^; *P* = 0.0057).

#### 3.2.2 Bed rest versus upright baseline

After 3 days of bed rest, total cerebral blood flow on average did not fall below values measured in the upright posture (seated baseline vs. 72 h, *P* = 0.3312). Blood flow in the internal carotid and VA likewise did not fall below values measured in the upright posture after bed rest (ICA, *P* = 0.5834; VA, *P* = 0.2863), while MCAv was slightly decreased compared to the seated position (seated vs. 72 h, 52.8 ± 15.1 vs. 49.3 ± 12.3 cm s^−1^; *P *= 0.0395).

An overview of changes in cerebral blood flow over the 3 days of bed rest is presented in Tables 1 and 2 and Figure 2.

**TABLE 2:** Internal carotid and vertebral artery blood flow before (baseline), during (24 and 48 h) and after (72 h) bed rest.

Parameter	Baseline		3 days’ bed rest		
	Seated 90°	Supine	24 h	48 h	72 h
ICA blood flow (mL min^−1^)	345 ± 140^**^	427 ± 146	387 ± 122^*^	363 ± 120^*^	337 ± 118^**^
ICA velocity (cm s^−1^)	30.8 ± 6.1^*^	34.4 ± 6.0	33.4 ± 6.8	33.4 ± 6.0	30.5 ± 5.5^*^
ICA diameter (cm)	0.485 ± 0.08^*^	0.511 ± 0.09	0.496 ± 0.08^*^	0.479 ± 0.08^**^	0.482 ± 0.09^**^
ICA conductance (mL min^−1^ mmHg^−1^)		5.32 ± 1.78	4.67 ± 1.29^*^	4.49 ± 1.49^*^	4.2 ± 1.47^**^
VA blood flow (mL min^−1^)	100 ± 54	112 ± 59	94 ± 45	102 ± 54	90 ± 45^*^
VA velocity (cm s^−1^)	16.7 ± 5.5	17.9 ± 5.0	15.7 ± 4.3^*^	16.6 ± 5.5	14.8 ± 4.6^**^
VA diameter (cm)	0.344 ± 0.06	0.353 ± 0.06	0.346 ± 0.06	0.35 ± 0.06	0.35 ± 0.06
VA conductance (mL min^−1^ mmHg^−1^)		1.46 ± 0.88	1.16 ± 0.57^*^	1.32 ± 0.85	1.16 ± 0.71^*^

*Note*: Values are means ± SD. *n* = 17. *P*‐values of changes are all compared to supine posture. *P < 0.05, **P < 0.001; for detailed *P*‐values see ‘Results’ section and figures. Abbreviations: ICA, internal carotid artery; VA, vertebral artery.

### 3.3 Changes in $ET_{CO_{2}}$ and cardiovascular parameters after 3 days of bed rest

#### 3.3.1 Supine baseline

During bed rest, $ET_{CO_{2}}$ showed a similar trend to blood flow in the vertebral artery with a tendency to decrease only after 72 h (supine vs. 72 h, 42 ± 3 to 40 ± 3 mmHg; *P* = 0.0803). Mean arterial blood pressure remained at values similar to the supine posture throughout the study (supine vs. 72 h, *P* = 0.9707).

#### 3.3.2 Upright baseline

No difference was observed for $ET_{CO_{2}}$ between the seated baseline and 3 days of bed rest (*P* = 0.3913). MAP at the level of the brachial artery was lower during bed rest compared to the seated posture (*P* = 0.0021).

An overview of haemodynamic parameters over 3 days of bed rest is presented in Table 1, 2 and Figure 3.

## 4 DISCUSSION

On changing posture from the upright to the supine position and over 3 days of bed rest, three main findings were observed. (1) Total cerebral blood flow is lower in the upright posture, but predominantly due to changes in blood flow to the anterior cerebral circulation. (2) Total cerebral blood flow decreases over 72 h of bed rest compared to the supine position, with blood flow decreasing in both the anterior and posterior cerebral circulations, though not below levels normally experienced in the upright position during daily life. (3) The mechanism(s) responsible for changes in cerebral blood flow are not clearly explained by a decrease in CPP and/or hypocapnia as changes in arterial pressure and $ET_{CO_{2}}$ do not precisely mirror regional changes in blood flow through the ICA and VA.

### 4.1 Effects of changing from the upright to the supine posture on Earth

Similar to previous studies (Alperin et al., 2005; Sato Fisher, et al., 2012), we observed total cerebral blood flow to be lower in the upright than in the supine posture. This reduction is likely mainly due to vasoconstriction in the anterior cerebral circulation, as blood flow in the ICA and blood velocity in the middle cerebral artery were lower, but blood flow in the vertebral artery was preserved. Together, these data suggest regional differences in the regulation of cerebral blood flow to orthostatic stress. Sato, Fischer, et al. (2012) noted analogous changes to ours between the supine position and a 60° head‐up tilt. They concluded that, as the vertebral artery provides blood flow to the medulla oblongata, which includes important cardiorespiratory and vasomotor control centres (Tatu et al., 1996), the posterior circulation is likely to be more important for maintenance of cardiorespiratory regulation during orthostatic stress and therefore more tightly regulated. Kay & Rickards (2016) found similar results, noting that posterior cerebral artery velocity is preserved during the application of lower‐body negative pressure in individuals tolerating high levels (and thus also tolerating higher magnitudes of central hypovolaemia).

While the mechanism(s) responsible for this regional difference is surprisingly unclear, changes in CPP are unlikely to be the culprit. Mean arterial blood pressure may at first glance be a likely candidate, as, measured at the brachial artery, it does increase significantly with the change from a supine to an upright position. However, it is worth noting that CPP (MAP minus intracranial pressure) needs to be calculated at the level of the brain, and not at the brachial artery. Previous data have shown that after accounting for the decrease in blood pressure due to the hydrostatic gradient from the arm to the brain, alongside the posture‐related change in intracranial pressure, CPP remains constant despite changes in posture (Petersen, Petersen, et al., 2016. This stability is generated by orthostatic stress causing similar changes in both MAP and intracranial pressure at the brain level (Lawley et al., 2017; Petersen, Jaekel, et al., 2016).

As $ET_{CO_{2}}$ was significantly decreased in the seated position, posture‐related hypocapnia seems a logical explanation for blood flow differences, and a divergence in CO_2_ reactivity between the anterior and posterior cerebral circulation might explain regional differences to orthostatic stress. However, literature is divided on the effect of $ET_{CO_{2}}$ on the anterior cerebral circulation. Immink and colleagues found the contribution of $P_{\mathrm{aC}O_{2}}$ to the reduction in MCAv to be transient, as, during a 5‐min 70° upright tilt, isocapnic clamping to a supine level only prevented a reduction of MCAv in the first minute (Immink et al., 2009). In contrast, a recent study showed that clamping $ET_{CO_{2}}$ at isocapnic levels leads to the maintenance of ICA blood flow and MCA velocity during the application of lower‐body negative pressure (Anderson et al., 2023). Moreover, while CO_2_ reactivity is generally blunted in the vertebro‐basilar circulation (Sato, Sadamoto, et al., 2012; Skow et al., 2013; Willie et al., 2012), a blunted CO_2_ reactivity may not be a clear explanation for regional differences as both our study and others (Sato, Fisher, et al., 2012) observed no difference in VA blood flow between positions, rather than a ‘blunted’ response that would be suggestive of reduced sensitivity. Other mechanistic factors, such as cardiac output and postural‐related haemoconcentration (Jacob et al., 2005) with a subsequent elevation in arterial oxygen content, likely explain this postural decrease in ICA blood flow, although why VA blood flow is not similarly affected is a question for future research.

### 4.2 Effects of 3 days of bed rest on global cerebral blood flow

Several short‐term (Frey et al., 1993; Marshall‐Goebel et al., 2016) and middle‐ to long‐term bed rest studies (Ogoh et al., 2020; Sun et al., 2005) have investigated cerebral blood flow and found a decline compared to the supine posture. In the present study, total cerebral blood flow showed a consistent decrease over all 3 days without reaching an obvious ‘steady‐state’. However, total cerebral blood flow did not fall significantly below the upright baseline at any time point. While a few studies have reported that cerebral blood velocity is unchanged following bed rest (Jeong et al., 2014, 2012; Pavy‐Le Traon et al., 2002), differences in study protocols may explain the discrepancy. First, the aforementioned studies only used transcranial Doppler ultrasound to indirectly assess brain blood flow from the velocity of blood within the middle cerebral artery, which does not take into account changes in intracranial artery diameter. However, in our opinion this technical difference is unlikely to provide a simple explanation: we also used transcranial Doppler to supplement our volumetric flow data, and it generally showed comparable changes to blood flow measured in the ICA. Second, many previous bed rest studies allowed head elevation (e.g., pillow use). While the addition of a pillow may seem minor, we have previously shown that its use causes significant gravitational unloading of intracranial pressure (Lawley et al., 2017), which may impact the mechanism(s) responsible for the reduction in cerebral blood flow and contribute to the discrepancy of these studies’ results. While a reduction in cerebral blood flow is clear with bed rest, experiments performed in true microgravity have varying outcomes. This is potentially due to the adaptation process in zero gravity and/or differences in exercise training performed by astronauts over different missions, which may act as a countermeasure against microgravity‐ and/or physical inactivity‐induced reductions in cerebral blood flow. Nevertheless, while one study noted a tendency for cerebral blood flow velocity to be lower (Herault & Poliakov, 2000) during 5 months of spaceflight, the majority of evidence suggests that cerebral blood flow is reduced early during space flight—similar to the upright posture via tilt or lower body negative pressure on Earth—and thereafter increases back to normal levels within a few weeks (Arbeille et al., 2001; Iwasaki et al., 2007). This is a strikingly similar situation to the current study which, to the best of our knowledge, is the only study to have collected control measurements in both the supine and upright posture, whereby cerebral blood flow during bed rest on average did not decrease below the upright posture.

While cerebral blood flow did not fall below the baseline values in a seated position, this comparison lacks the fluctuations in blood flow caused by postural changes during daily life. A simplified calculation may be used as a demonstration of blood flow over 24 h: assuming that humans on average spend about 16 h per day upright and 8 h in a supine position, we calculated a 24‐h mean (=16×seated+8×supinebaseline) and compared this 24‐h baseline to the last day of bed rest. Interestingly, cerebral blood flow after bed rest was lower than this calculated average (calculated 24 h vs. 72 h, 969 ± 292 to 853 ± 253 mL min^−1^; *P* = 0.0066). It is worth noting that this calculation does not take into account changes in cerebral blood flow during physical activity and the decrease normally observed during sleep (Klingelhöfer et al., 1995). However, at present, there is no reason to believe that similar changes in cerebral blood flow would not also occur if physical activity or sleep is performed while on bed rest. Thus, while not perfect, the calculation illustrates the importance of future studies to try to account for both changes in posture and the amount of time spent in that position when evaluating bed rest data.

### 4.3 Effects of 3 days of bed rest on regional cerebral blood flow

On a regional level, Marshall‐Goebel et al. (2016) saw a reduction in blood flow in the ICA with short‐term (3 h) progressive (−6, −12 and −18°) HDT. Extending these data to 3 days of bed rest, we observed that blood flow in both the anterior (internal carotid and middle cerebral arteries) and posterior (vertebral artery) cerebral circulations declined compared to the supine posture. While ICA blood flow was decreased from the first day of bed rest, blood flow in the VA only significantly decreased on day 3. Similar trends have also been noted via photon emission computed tomography after 5 days of dry immersion (Guillon et al., 2021) and while decreases in ICA or VA blood flow could not be detected over 3 days of dry immersion (Ogoh et al., 2017), potentially due to a small sample size, conductance in both arteries did decrease, highlighting cerebral vasoconstriction.

Interestingly, during long‐term bed rest, regional variations in cerebral perfusion once again become apparent. Ogoh et al. (2020) observed a clear reduction in ICA conductance after 30 days of bed rest (supine, 4.6 ± 2.21 vs. 30 days bed rest, 3.49 ± 1.35 ml min^−1^ mmHg^−1^). In contrast, vertebral artery conductance was unchanged throughout the campaign. Data for vascular conductance mirrored changes in cerebral blood flow (Ogoh et al., 2020). Similarly, vertebral artery conductance in the current study decreased statistically after only 72 h of bed rest, while conductance in the ICA was decreased starting with the first measurement after 24 h, then further decreasing throughout the study.

### 4.4 Changes in $ET_{CO_{2}}$ and MAP as mechanism(s) causing the decrease in cerebral blood flow with bed rest

Surprisingly few studies have performed experimental interventions to isolate the mechanism(s) responsible for the decrease in cerebral blood flow with bed rest. While most observational studies measure blood pressure, relatively few have reported $ET_{CO_{2}}$, which is a potent stimulus for changes in cerebral vascular tone. In the current study, $ET_{CO_{2}}$ was slightly reduced during bed rest. Therefore, hypocapnia could have contributed to the reduction in cerebral blood flow. While this is a tempting hypothesis, two observations must be noted. First, the general trend that blood flow in the internal carotid and middle cerebral arteries remained reduced over time does not reflect the dynamics observed in end‐tidal carbon dioxide (Figures 2 and 3). However, fluctuations similar to those in $ET_{CO_{2}}$ were observed in vertebral blood flow (Figure 2). Second, previous data have shown that blood flow in the ICA remained reduced for at least up to 30 days of bed rest, at which point $ET_{CO_{2}}$ was normalized (supine, 38.7 ± 1.4 vs. 30 days bed rest, 39.3 ± 2.4 mmHg).

While not measured in the current study, the fall in plasma volume is similar (∼10%) between the supine and upright posture (Jacob et al., 2005), and between the supine posture and after several days of bed rest (Greenleaf et al., 1989; Iwasaki et al., 2004). The magnitude of reduction in plasma volume, and thus potential haemoconcentration, mirrors data from the current study where blood flow to the brain was reduced similarly between the upright posture and 3 days of bed rest. Unfortunately, to date, only one study has performed an experimental manipulation of plasma volume (exercise training and/or dextran infusion (volume loading)) during bed rest (18 days) alongside estimates of cerebral blood flow (Jeong et al., 2014). In this study, resting MCAv was reduced with bed rest (pre‐bed rest, 64.9 vs. post‐bed rest, 62.3 cm s^−1^) and quantitatively normalized after dextran infusion (post‐dextran, 65.6 cm s^−1^). Yet statistical significance was not apparent, likely due to both the small sample size and the fact that this study was not designed to offset the bed rest‐induced increase in haemoglobin concentration (Ryan et al., 2016) and oxygen carrying capacity, but to normalize cardiac filling pressures. Interestingly, Iwasaki et al. (2020) recently examined MCAv in astronauts pre‐ and post‐space flight and noticed an increase in MCAv shortly after returning to Earth. They hypothesized this increase occurred due to a decrease in haemoglobin concentration and thus arterial oxygen content owing to acute infusions and oral volume loading after landing (Iwasaki et al., 2020). Therefore, posture‐related haemoconcentration (i.e., increased arterial oxygen content) may provide a simple explanation as to why cerebral blood flow is lower in the upright position, also during bed rest or short‐term microgravity without countermeasures, and why it is normalized over several weeks of space flight. It is also important to consider that a fall in plasma volume will increase whole blood viscosity and independently reduce blood flow to the cerebral circulation. Yet, an increase in viscosity also causes an increase in shear stress and NO‐mediated vasodilatation (Hoiland et al., 2019). Ultimately it seems these factors are balanced out, and several studies have concluded that blood viscosity plays a limited role in regulating cerebral blood flow (Brown & Marshall, 1985; Brown et al., 1985), supporting a major role of oxygen content.

Future research could also examine why the vertebro‐basilar circulation seems more sensitive to $ET_{CO_{2}}$ than the anterior cerebral circulation in the supine posture (Sato, Sadamoto, et al., 2012; Willie et al., 2012) and during acute bed rest, but seems disassociated during long‐term orthostatic stress. Importantly from our point of view, a reduction in CPP is an unlikely mechanism as MAP remained unchanged relative to the supine posture during the 3 days of bed rest and the very slight increase in intracranial pressure is normalized even within 24 h of bed rest (Lawley et al., 2017).

## 5 LIMITATIONS

A major limitation of this study is its observational nature. However, the combination of several imagining modalities (duplex and transcranial Doppler) combined with haemodynamic monitoring over several time points lends a high degree of confidence to the data and the ability to develop future mechanistic targets for interventional studies. A second limitation is that we measured $ET_{CO_{2}}$ and not $P_{\mathrm{aC}O_{2}}$, which may overestimate the degree of hypocapnia during orthostatic stress due to ventilation/perfusion mismatch (Zhang & Levine, 2007), although Tymko and colleagues found no difference in $P_{\mathrm{ETC}O_{2}}$ and $P_{\mathrm{aC}O_{2}}$ in normoxic conditions (Tymko et al., 2016). Statistical power is always a limiting factor in small bed rest studies. In the current study, changes in blood flow through the vertebral artery during bed rest were smaller in magnitude compared to the ICA, but consistently lower albeit not always reaching statistical significance. It is likely that changes in posture and bed rest cause very subtle changes to blood flow to the posterior cerebral circulation and its influence needs to be further investigated. Third, we combined measurements from a −6° head down tilt bed rest with a second part of the study taking place in a horizontal position. However, the horizontal position was deemed a NASA standard at the time and was adopted for that reason, with the assumption that the outcome for cardiovascular parameters would not differ. Despite the gravitational gradient being slightly different between the heart and the brain in the head down tilt position, we observed similar decreases in cerebral blood flow during bed rest. Finally, bed rest studies and quiet upright rest are not directly analogous to spaceflight and/or daily life. This lack of model precision is because (1) bed rest does not eliminate the gravitational force acting in a sagittal axis (*G_x_*‐force) and (2) sitting quietly in the upright posture and lying down in bed does not reflect a human's typical day on Earth or in space. In the awake state on Earth and/or in space humans perform multiple seated–standing transitions and exercise, which are not reflected in the measurements (or calculations, i.e., a theoretical 24 h CBF) obtained in this study. Nevertheless, the headward fluid shift together with the factors of confinement makes bed rest a reliable model for changes in haemodynamic systems associated with living in microgravity (Green, 2006; Hinghofer‐Szalkay, 2011). Moreover, it provides a realistic model for bed rest in patient populations on Earth.

## 6 CONCLUSION

Compared to lying supine, cerebral blood flow decreases in the upright posture, predominantly in the anterior cerebral circulation. With bed rest, cerebral blood flow also decreases over time, and to a similar extent, but in both the anterior and posterior cerebral circulations. While this decrease was on average not below that typically experienced in the upright position during daily life, it was lower than a 24‐h mean calculated from both seated and supine baseline values. Changes in MAP and/or $ET_{CO_{2}}$ do not appear to be clear explanations for these observations. Future research is needed to ascertain if the decrease in cerebral blood flow is linked to cognitive impairments, or is a simple reflex due to posture‐related haemoconcentration and an increase in arterial oxygen‐carrying capacity that is typically observed during bed rest.

## AUTHOR CONTRIBUTIONS

Benjamin D. Levine, Justin S. Lawley, Christopher M. Hearon Jr., and Katrin A. Dias designed the experiment. Benjamin D. Levine, Justin S. Lawley, Christopher M. Hearon Jr., Katrin A. Dias, Gautam Babu, Sylvan L. J. E. Janssen, and Satyam Sarma performed the measurements. All authors discussed the results and worked on the manuscript. All authors have read and approved the final version of this manuscript and agree to be accountable for all aspects of the work in ensuring that questions related to the accuracy or integrity of any part of the work are appropriately investigated and resolved. All persons designated as authors qualify for authorship, and all those who qualify for authorship are listed.

## CONFLICT OF INTEREST

The authors declare no conflict of interest.

## Data Availability

All original data will be made available by reasonable request.
